# Crystallographic structure of wild-type SARS-CoV-2 main protease acyl-enzyme intermediate with physiological C-terminal autoprocessing site

**DOI:** 10.1038/s41467-020-19662-4

**Published:** 2020-11-18

**Authors:** Jaeyong Lee, Liam J. Worrall, Marija Vuckovic, Federico I. Rosell, Francesco Gentile, Anh-Tien Ton, Nathanael A. Caveney, Fuqiang Ban, Artem Cherkasov, Mark Paetzel, Natalie C. J. Strynadka

**Affiliations:** 1 Department of Biochemistry and Molecular Biology and Centre for Blood Research, The University of British Columbia, Vancouver, BC Canada; 2 Department of Molecular Biology and Biochemistry, Simon Fraser University, Burnaby, BC Canada; 3 Vancouver Prostate Centre, The University of British Columbia, Vancouver, BC Canada

**Keywords:** Enzyme mechanisms, Drug discovery, SARS-CoV-2, X-ray crystallography

## Abstract

Severe Acute Respiratory Syndrome Coronavirus 2 (SARS-CoV-2), the pathogen that causes the disease COVID-19, produces replicase polyproteins 1a and 1ab that contain, respectively, 11 or 16 nonstructural proteins (nsp). Nsp5 is the main protease (M^pro^) responsible for cleavage at eleven positions along these polyproteins, including at its own N- and C-terminal boundaries, representing essential processing events for subsequent viral assembly and maturation. We have determined X-ray crystallographic structures of this cysteine protease in its wild-type free active site state at 1.8 Å resolution, in its acyl-enzyme intermediate state with the native C-terminal autocleavage sequence at 1.95 Å resolution and in its product bound state at 2.0 Å resolution by employing an active site mutation (C145A). We characterize the stereochemical features of the acyl-enzyme intermediate including critical hydrogen bonding distances underlying catalysis in the Cys/His dyad and oxyanion hole. We also identify a highly ordered water molecule in a position compatible for a role as the deacylating nucleophile in the catalytic mechanism and characterize the binding groove conformational changes and dimerization interface that occur upon formation of the acyl-enzyme. Collectively, these crystallographic snapshots provide valuable mechanistic and structural insights for future antiviral therapeutic development including revised molecular docking strategies based on M^pro^ inhibition.

## Abstract (web summary)

The SARS-CoV-2 main protease (M^pro^) is one of two cysteine proteases essential for viral replication. Here, the authors determine the crystal structure of an M^pro^ acyl intermediate with its native C-terminal autocleavage sequence and the structure of a product bound active site mutant (C145A), which are of interest for antiviral drug development.

## Introduction

SARS-CoV-2 main protease (M^pro^) is one of two cysteine proteases necessary for viral replication and assembly, with analogous functional counterparts in earlier SARS-CoV-1 and Middle Eastern Respiratory Syndrome (MERS) coronavirus variants^1^. SARS-CoV-2 M^pro^ is 306 residues in length corresponding to residues 3264–3569 within the large polyproteins pp1a or pp1ab (generated by a ribosomal frameshift during translation). It is denoted by enzyme commission number EC: 3.4.22.69 within the Merops Database Classification subclan PA(C) and family C30. Initial structures of SARS-CoV-1 M^pro^ (~96% identical to SARS-CoV-2 M^pro^) showed a dimer with each protomer composed of a double-barreled catalytic region, with structural similarity to 3C protease found in picornaviruses (and thus its alternate name 3C-like protease 3CL^pro^), that is followed by an all α-helical domain^2,3^. The structures also suggested the enzyme employs a cysteine (Cys145) side chain thiolate as a nucleophile in the presumed initial acylation step of peptide bond cleavage, potentially assisted by an adjacent histidine (His41) in the enzyme active site (see Supplementary Fig. 1a for reaction schematic). M^pro^ cleavage of pp1a and pp1ab at the 11 sites, including autoprocessing sites at its own N- and C-termini, releases nonstructural proteins (nsp) 4–16. SARS-CoV-1 M^pro^ has been shown to proteolyze dodecapeptides spanning each of the 11 processing sites (Supplementary Fig. 1b), cleaving after glutamine in the consensus sequence (**P2:Leu**/Met/Phe/Val)-**P1:Gln**↓-(**P1′:Ser/Ala/**Gly/Asn) with the two peptides corresponding to the N- and C-terminal M^pro^ autocleavage sites having the highest efficiency^4^.

Although crystallographic structures of SARS-CoV-2 M^pro^ are compounding weekly in the literature, in native forms^5–7^ and with various bound chemical fragments^5^ or inhibitors^7–10^, a missing link for SARS-CoV-2 M^pro^ and indeed its SARS-CoV-1 and MERS-CoV relatives, remains the lack of atomic resolution information for key intermediary mechanistic steps with native active site and physiological substrate(s). To that end, in this paper, we present the structure, at 1.95 Å resolution, of the wild-type acyl-enzyme intermediate of SARS-CoV-2 M^pro^ covalently bound to its natural autocatalytic processing site at its C-terminus. Capture of this intermediate provides atomic details of the acyl-enzyme coordination geometry and stabilization, the surrounding solvation/desolvation, as well as the underlying substrate specificity determined by side chain type and orientation of the P1–P6 residues—SGVTFQ—with those of the complimentary pockets in the M^pro^ active site. Further, a product complex of the same substrate, captured at 2.0 Å resolution using a Cys145Ala mutation, is also presented, providing further mechanistic and atomic information to inform future therapeutic design.

## Results

### Purification of an active M^pro^ dimer

**Fig. 1:**
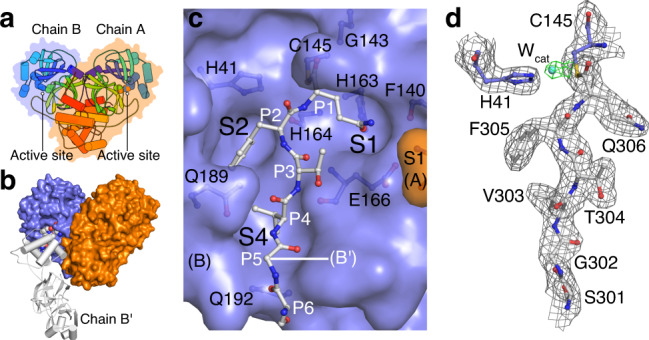
Wild-type SARS-CoV-2 M^pro^ acyl-enzyme intermediate structure at 1.95 Å resolution. **a** Overview of M^pro^ dimer. Each protomer colored spectrally (N-terminus blue to C-terminus red). A transparent molecular surface is shown around each protomer (chain A—orange, chain B—blue). **b** M^pro^ structure determined here shown in molecular surface colored as in (**a**). A symmetry-related chain in the crystal lattice (B′, white) directs its C-terminal six residues into the substrate binding groove of chain B (Ser301–Gln306 shown in CPK space filling representation). **c** Substrate binding groove (blue surface) of chain B with covalently bound C-terminal P1–P6 residues of B′. The N-terminus of chain A (Ser1, the so-called N-finger, orange surface) provides structural support for the S1 pocket of chain B. The side chains of the catalytic residues Cys145, His41, and residues that make direct hydrogen bonds to substrate are shown. **d** 2mF_o_-DF_c_ electron density contoured at 1.0*σ* around the chain B′ C-terminus clearly reveals the thioester bond. Electron density for *W*_cat_ adjacent to the thioester carbonyl carbon shown in green, also contoured at 1.0*σ*. A simulated annealing OMIT map for the bound substrate is shown in Supplementary Fig. 5a.

Recombinant SARS-CoV-2 M^pro^ with native N- and C-termini^11^ was overexpressed and purified with slight modifications of previous protocols^9^. We also produced the catalytic mutant C145A and a mutant that impacts dimerization, P9T, with similar protocols, see “Methods”. Biological small-angle X-ray scattering (bioSAXS) and SEC-MALS demonstrate the wild-type protein is exclusively a dimer across a range of protein and salt concentrations, while the P9T mutant is predominantly monomeric (Supplementary Figs. 2 and 3). Based on the published analytical ultracentrifugation results, SARS-CoV-2 M^pro^ is known to exhibit a high propensity for dimerization with a *K*_D_ ~ 2.5 μM (ref. ^7^). Dimerization, with the two protomers associated at right angles to form a heart-shaped complex (Fig. 1a, b), has been shown to be critical for efficient catalytic activity in SARS-CoV-1 (ref. ^12^), with the interface interactions required for correct active site structure, including notably the N-terminus (N-finger, Ser1) of each protomer stabilizing the S1 substrate binding pocket of its neighboring protomer^2,3^ (Fig. 1c, orange surface). The wild-type M^pro^ preparation used for our structural analysis is active in a FRET-based assay with enzymatic parameters and inhibition by the antineoplastic agent carmofur (IC_50_ value of 1.8 ± 0.3 μM) consistent with those previously determined^8^ (Supplementary Fig. 4). As also observed elsewhere^13^, the determined Hill coefficient was greater than one, indicating positive cooperativity. Possible explanations for this, which future studies may unravel, include allosteric communication between the two active sites within the dimer upon substrate binding or, alternatively, a substrate-induced dimerization. Mutation of the catalytic cysteine to alanine (C145A) abolishes activity, while the dimerization defective P9T (with native active site) lowers the catalytic efficiency by >50 fold (Supplementary Fig. 4c).

### Crystallographic determination of M^pro^ in complex with its C-terminal autocleavage sequence

Using X-ray crystallography, we have captured two unique structures of SARS-CoV-2 M^pro^ in complex with its C-terminal autocleavage site in *trans*, representative of distinct enzyme mechanistic states. First, an acyl-enzyme intermediate with the C-terminal residues bound in the active site of a neighboring dimer and Gln306 covalently bound to catalytic Cys145 in the wild-type protein and, second, a product-like form with the same C-terminal autocleavage sequence observed bound non-covalently in the active site of a catalytically inactive C145A mutant.

**Fig. 2:**
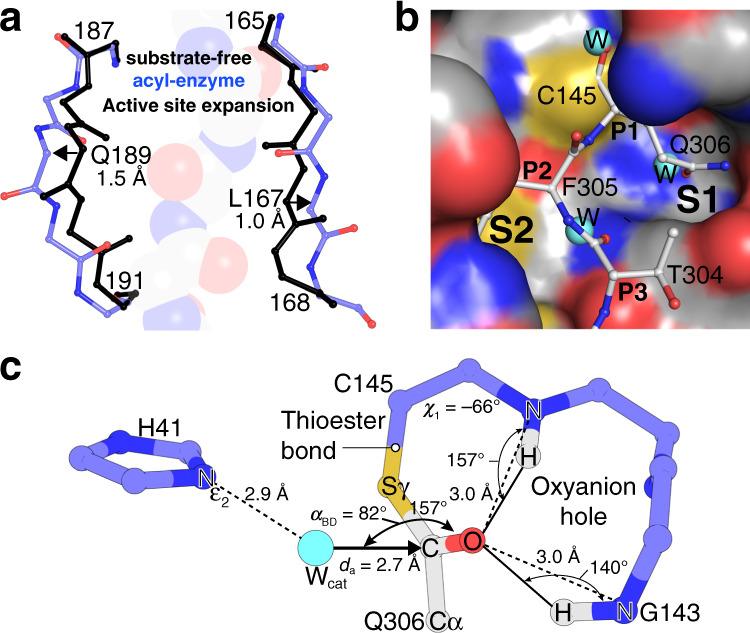
Comparison of wild-type acyl-enzyme intermediate and substrate-free M^pro^ structures. **a** Superposition of the substrate-free (black) and acyl-enzyme (blue) forms reveals changes in the substrate binding groove width. The main chain atoms for bound B′ substrate are shown as transparent van der Waals spheres. **b** Molecular surface of wild-type M^pro^ with three-ordered water molecules (cyan spheres). Superposition of the acyl-enzyme structure shows these waters are coincident with oxygen atom positions and will be displaced upon substrate binding. **c** Analysis of the wild-type acyl-enzyme active site reveals a potential deacylating water (catalytic/nucleophilic–*W*_cat_) approaching the Re*-*face of the thioester. Ball and stick diagram depicting the geometry and atomic interactions of the thioester linkage between the Sγ of Cys145 and main chain carbonyl carbon of substrate Gln306. The trigonal planar nature of the thioester group, defined by atoms Cα, C, and O of Gln306, and Sγ of Cys145 is shown as is the *χ*_1_ dihedral angle (defined by atoms N, Cα, Cβ, and Sγ). The oxyanion hole hydrogen bond distances and angles are also labeled. Proposed deacylating water (*W*_cat_) shown as a cyan sphere. *α*_BD_ is the Bürgi–Dunitz angle (*W*_cat_-*C*=O) and *d*_a_ the attack distance.

**Fig. 3:**
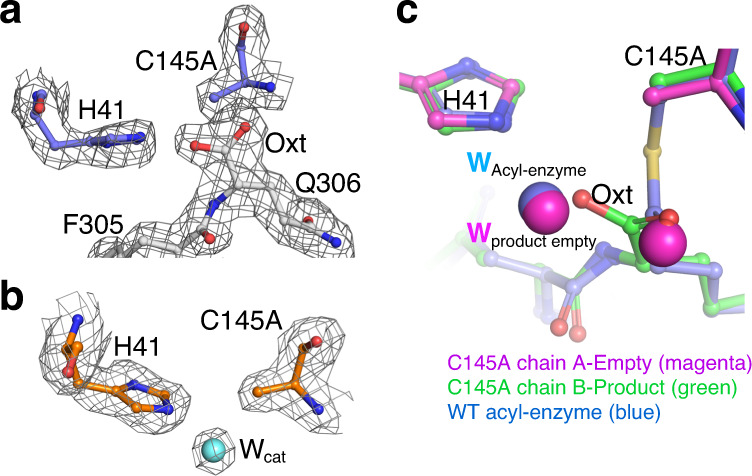
C145A SARS-CoV-2 M^pro^ product complex at 2.0 Å resolution. **a** 2mF_o_-DF_c_ electron density (contoured at 1.0*σ*) in chain B of the C145A mutant shows presence of the bound C-terminal product of symmetry-related molecule B′. Also see Supplementary Fig. 5b. **b** 2mF_o_-DF_c_ electron density (1.0*σ*) of empty protomer, chain A, of the same C145A mutant structure shows presence of a highly ordered water molecule hydrogen bonded to Nε2 of His41, consistent with a general base role of the latter and coincident in position with the *W*_cat_ weakly observed in the acyl-enzyme complex as in **c** and Fig. 1c. **c** Superposition of the product chain A (empty binding site; magenta) and chain B (product bound; green) with the acyl-enzyme (chain B; blue).

Wild-type and C145A M^pro^ were crystallized at pH 6 in space group *C*2 with isomorphous unit cell dimensions (Supplementary Table 1). For both structures, the asymmetric unit is composed of an M^pro^ dimer with crystal packing orienting the C-terminus of one monomer (chain B′) proximal to the active site of a symmetry-related monomer (chain B; Fig. 1b). In the mature enzyme (residues 1–306), the C-terminal autocleavage sequence Ser301–Gln306 packs at the dimerization interface as observed in chain A; however, in chain B it is instead rotated almost 180° toward domain III and inserted into the neighboring active site, occupying the S6–S1 substrate binding pockets (Fig. 1c). This results in one protomer with substrate bound and one empty in each dimer pair. For the wild-type acyl-enzyme intermediate complex, there is clear continuous density showing the carbonyl carbon atom of the C-terminal Gln306 covalently bound to the sulfur atom of catalytic Cys145 (Fig. 1d and Supplementary Fig. 5a). The C-terminal autocleavage site binds within the substrate binding groove in an extended conformation (Figs. 1c and 2a, b), making antiparallel β-sheet, as well as side chain-mediated hydrogen bond interactions with residues 164–166 of β-strand 12 (see Supplementary Fig. 6 for numbering) on one side, and with residues 189–191 of the ~15 residue loop linking domains II and III on the other (Fig. 2a and Supplementary Fig. 7a). For the C145A mutant product-like complex, the C-terminus binds in the same extended manner, forming analogous main chain and side chain interactions (Supplementary Fig. 7b). Well-ordered electron density unambiguously confirms the alanine mutation and presence of terminating main chain carboxylate oxygens (Fig. 3a and Supplementary Fig. 5b).

### Characterization of the M^pro^ acyl-enzyme intermediate complex with physiological substrate

The 1.95 Å resolution structure of the SARS-CoV-2 M^pro^ provides atomic details of the fully resolved acyl-enzyme intermediate state central to the catalysis of pp1a/ab processing during viral maturation and pathogenicity. The electron density clearly defines the stereochemistry of the thioester bond formed between the Cys145 side chain γ-sulfur atom and the carbonyl carbon of its (self) substrate at the residue preceding the scissile bond position Gln306 (P1) as trigonal planar (Sγ-C-O-Cα) and with a Cys145 *χ*_1_ angle of −66°. The carbonyl oxygen of the intermediate is stabilized by bifurcated hydrogen bonds with the main chain nitrogens of Cys145 and Gly143 at distances for both of 3.0 Å (Fig. 2c and Supplementary Fig. 7a), providing an ideal oxyanion hole interaction. The closest approach of the thioester sulfur of Cys145 to the potential general acid/base His41 is a distance of 3.7 Å to its Nε2, and at an angle not in keeping with a direct hydrogen bond. The general disposition of the Cys145 Sγ to substrate P1 carbonyl delineate a Re-face attack in the acylation step (Fig. 2c). The His41 imidazole is packed between the side chains of Pro39 and Met49, with Nδ1 hydrogen bonding to a highly ordered, multi-coordinated (His164, Asp187 side chains), and buried water molecule, previously proposed to play a role in regulating the protonation state of His41 in SARS-CoV-1 via QM/MM calculations^14^. The solvent accessible surface of the His41 imidazole Nε2 calculated by Areaimol^15^ is almost fully buried, with a value of 1.4 Å^2^, as compared to the free active site, 7.9 Å^2^, a factor likely influencing the p*K*_a_, protonation state, and potential role in catalysis.

Anchored by the covalent thioester bond to Cys145, oxyanion hole coordination, and extended β-sheet hydrogen bonding of substrate and active site, the P1–P6 (QFTVGS) specificity determinants are accommodated within the inward facing enzyme cleft pockets S1, S2, and S4 (Fig. 1c), providing multiple stabilizing noncovalent interactions (Supplementary Fig. 7a) and collectively 512 Å^2^ of buried enzyme surface. Notably, the presence of a phenylalanine in the P2 position results in a wider binding pocket compared to the empty active site forms, created by shifts of the side chains of Met165 and Gln189 to accommodate, with the side chain amide of the latter redirecting to form stabilizing hydrogen bonds with the P4 main chain atoms (Fig. 1c and Supplementary Fig. 7). Overlap of the acyl-enzyme intermediate structure with the wild-type substrate-free structure (that determined here, as well as the highest resolution published structure PDB 6YB7) shows that the binding of substrate results in a significant outward shift and increase of overall width of the substrate binding groove (Fig. 2a). Residues making up the outer edge of the binding site adjust up to 1.5 Å on one side (residues 187–191) and up to 1.0 Å on the other side (residues 165–168), both regions which directly bind substrate, suggesting an unusual expansion of the binding groove rather than constriction typical of most serine proteases^16^ is required for the C-terminal autoprocessing substrate to optimally fit into the M^pro^ active site. Interestingly, if the same overlap is done with the empty protomer (chain A) in the acyl-enzyme intermediate or product complex dimers, this expansion is only observed for residues 187–191 and to a lesser degree, suggesting possible allosteric communication between the two protomers of the active dimeric form upon substrate binding.

Important aspects of solvation/desolvation in formation of the acyl-enzyme intermediate are also interpreted from our data. Displacement of three highly ordered water molecules visible in the wild-type substrate-free structure occupy the position of the substrate carboxyl oxygen in the oxyanion hole, the Gln306 (P1) side chain oxygen, and Thr304 (P3) main chain carbonyl oxygen (Fig. 2b). These same highly ordered water molecules are typically observed in other SARS-CoV-2 M^pro^ structures, including the highest resolution structure yet reported at 1.25 Å (ref. ^5^; PDB 6YB7), although in that case a DMSO molecule is observed in the P1 site with the sulfoxide oxygen overlapping the water binding location. Notably, an additional, unique water position, in keeping with that of a deacylating water nucleophile, is observed in the acyl-enzyme intermediate structure, with weak but significant density (Fig. 1d). Positioned orthogonally with respect to the other atoms in the trigonal planar thioester group (Fig. 2c), the 1.95 Å resolution allows for a measurement of the approximate attack angle that the oxygen of this water (upon activation, the hydroxide anion, OH^−^) would take relative to the substrate carbonyl carbon. The angle, often termed the Bürgi–Dunitz angle (*α*_BD_)^17^, is defined by three atoms: the nucleophile (in this case a water oxygen O), the carbonyl carbon C, and the carbonyl oxygen O (O---C=O angle), with the generalized ideal falling near 107°. The putative deacylating water sits approximately equidistant between the Nε2 of His41 in the acyl-enzyme (2.9 Å), reinforcing its role as the activating general base, and the scissile carbonyl carbon (2.7 Å) and with a Bürgi–Dunitz angle as above of ~82°. Displacement of catalytic water molecules is a reoccurring theme in structure-based drug discovery and the observation of this water position in the context of the acyl-enzyme intermediate is an important advance in this regard (discussed further below).

Given the typically short-lived nature of the acyl-enzyme catalytic intermediate during proteolysis, capturing these has been historically challenging. There has been some previous success in characterizing acyl-enzyme species at the atomic level in serine proteases, but these experiments typically required some level of impairment to the enzyme^18–20^, substrate (non-hydrolyzable adduct, poor substrates, or inhibitors), and/or conditions^21–24^ for a stable acylation to be observed in the crystal structure. No prior examples in the classic cysteine protease families proper have been published, although a putative cysteine protease-like glutathione hydrolase acylated with glutathione substrate captured at pH 3 has been described^25^ (histidine base deprotonation highly disfavored at this extreme pH). Here, the *trans* acyl-enzyme complex of full-length, wild-type M^pro^ is observed in the crystallographic lattice with the endogenous P1–P6 C-terminal product of symmetry-related molecule B′ binding into the active site cleft of molecule B. The reaction the enzyme has catalyzed within the crystal is the reverse reaction, it is presented with the product (the free carboxylate of Gln306 (P1) from B′), and it has formed the acyl-enzyme by creating the thioester with the nucleophile Cys145 (see Supplementary Fig. 1a for reaction schematic). It is possible that the local effective concentration of the product as afforded by the crystal lattice has contributed to driving the reaction backward to the form the thioester. The crystals were grown at pH 6, theoretically not low enough to prevent a solvent accessible histidine (p*K*_a_ ~6.5) from functioning as a general base to activate a deacylating/nucleophilic water, but certainly disabling optimal activity (estimated kinetically at <50% in SARS-CoV-1 using a pentadecapeptide substrate spanning the C-terminal cleavage site^26^). Given the observed putative deacylating water, with appropriate distances of histidine base to water to thioester intermediate, we can only further speculate that the slightly less than optimal angle of attack by the nucleophilic water, ~82° instead of the theoretical optimum of 107°, as predicted by Bürgi–Dunitz could also contribute to the intermediate capture here.

### Crystallographic structure of the SARS-CoV-2 M^pro^ C145A product complex with physiological substrate

Capture of a well-ordered product complex in the catalytically impaired SARS-CoV-2 M^pro^ C145A mutant is clearly defined in the electron density maps (Fig. 3a and Supplementary Fig. 5b). One oxygen of the terminating carboxylate sits coincident with that of the carbonyl of the thioester acyl-enzyme intermediate structure, forming hydrogen bonded interactions with the oxyanion hole main chain nitrogens of 2.9 and 3.0 Å (Fig. 3c and Supplementary Fig. 7b). The second carboxylate oxygen is positioned to form a strong inline hydrogen bond interaction with His41 Nε2 (2.9 Å), again supporting a role of the latter in general base activation of a nucleophilic water to form such a product. In that context, the active site of the empty protomer in the C145A structure reveals electron density for five water molecules, including one not observed in the wild-type substrate-free protomer active site and lying completely coincident with the proposed deacylating water position in the acyl-enzyme intermediate above, only observed at even greater occupancy (Fig. 3b, c). The ordered water (*B*-factor = 30 Å^2^), is again positioned orthogonally with respect to the other atoms in the trigonal planar thioester group and with a near identical Bürgi–Dunitz angle as verified by superposition of the substrate-free active site of the C145A and native acyl-enzyme structures (Fig. 3c). We note a structure of SARS-CoV-1 M^pro^ C145A in a product complex with its C-terminal autocleavage site at 2.8 Å resolution has been published previously^27^; however, potentially due to the lower resolution a catalytic water was not observed in that case.

### Model of the SARS-CoV-2 M^pro^ enzyme–substrate complex

**Fig. 4:**
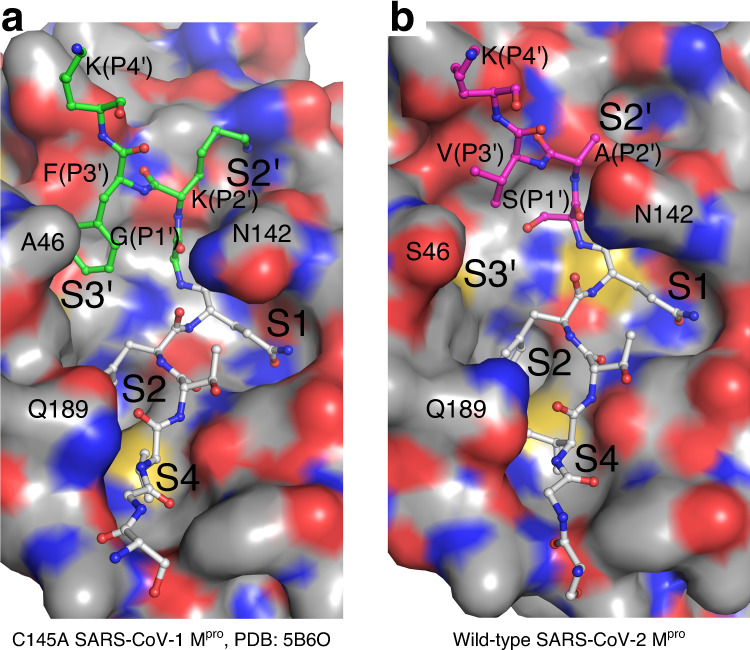
Modeling of the SARS-CoV-2 M^pro^ enzyme–substrate complex. **a** CPK molecular surface of SARS-CoV-1 C145A catalytic mutant ES complex (PDB 5B60), including C-terminal cleavage site P6–P4′ (P1′–P3′ with green carbons). **b** CPK molecular surface for the SARS-CoV-2 M^pro^ acyl-enzyme active site. The additional residues P1′–P4′ (magenta carbons) are modeled based on (**a**). Sequence alignment for all M^pro^ processing sites shown in Supplementary Fig. 1b. Note the identical sequence preceding the scissile bond between SARS-CoV-1 and -2 M^pro^, but divergence in P1′–P3′ (N-terminus of the subsequent nsp6). Despite these differences, the S1′–S3′ pockets observed in the SARS-CoV-2 M^pro^ acyl-enzyme active site are similar to that in (**a**), i.e., already preformed in the absence of P1′–P3′ (modeled here), and apparently not dependent on the binding of P2′. It is also evident from this panel that the P1′–P3′ side chains are not sterically matched to the S1′–S3′ pockets, perhaps an advantage in protein maturation.

An interesting aspect of viral polyprotein processing proteases, including SARS-CoV-1 and -2 M^pro^, are the requisite self-cleavage events to excise itself from precursor polyproteins result in a retained C-terminal product (P1–P6 as captured here) that could potentially act as a competitive inhibitor. By contrast, the N-terminal autoprocessing P1–P6 sequence (and C-terminal end of nsp4) departs after cleavage. Considering the M^pro^ consensus cleavage sequence (**P2:Leu**/Met/Phe/Val)-**P1:Gln**↓-(**P1′:Ser/Ala/**Gly), 9 out of the 11 in SARS-CoV-2 have a leucine in the P2 position, including the M^pro^ N-terminal (nsp4-nsp5) autoprocessing sequence (Supplementary Fig. 1b). Structures of SARS-CoV-1 M^pro^ in complex with the N-terminal sequence^28^ or of SARS-CoV-2 M^pro^ in complex with peptidomimetic inhibitors based thereon^8^ reveal the S2 subsite undergoes dramatic changes when it binds leucine in the P2 position, predominantly mediated by rearrangement of Met49 and Gln189, and surrounding regions. By contrast, the M^pro^ C-terminal autocleavage site in both SARS-CoV-1 and -2 is the only instance where there is a P2 phenylalanine which, when bound to the S2 subsite as observed here, maintains a more open conformation similar to the empty active site, albeit with movement of Met165 creating a deeper pocket. A structure of a SARS-CoV-1 M^pro^ C145A mutant in complex with its C-terminal prosequence at 2.2 Å resolution has been published previously^29^, representing an enzyme–substrate (ES) Michaelis-like complex (Fig. 4a). With Phe305 (P2) bound in the S2 subsite, Phe309 (P3′) was observed to bind in an adjacent complimentary pocket with mutation of Phe309 (P3′) reducing C-terminal cleavage. This multivalent binding interaction in S2 and the S3′ subsites was proposed to be needed to allow high-affinity binding of the C-terminal prosequence, supported by the 10× lower-affinity binding of the C-terminal P1–P4 sequence with Phe (P2) than the equivalent N-terminal sequence with Leu (P2)^29^. Although it was further suggested that Phe309 S3′ binding would be needed to order the adjacent S2 subsite, thus potentially avoiding autoinhibition by the retained post-cleavage mature C-terminal sequence, the structures here show that these subsites are equivalently in place in the substrate-free native, acyl-enzyme intermediate and product complexes (Fig. 4).

Based on the wild-type acyl-enzyme intermediate structure here, we have generated a model of the SARS-CoV-2 ES C-terminal complex, extending from P6–P1 the downstream residues encompassing P1′–P4′ (Fig. 4b). Although the P1′–P3′ positions in the SARS-CoV-2 C-terminal processing site are distinct compared to SARS-CoV-1 (SAV vs GKF, respectively), the structure of the S3′ subsite region is near identical (RMSD = 0.363 Å on 50 common atoms) with the only differences compared to SARS-CoV-1 an A46S substitution on one edge of the cavity that could provide, along with the Ser307 (P1′), van der Waals interactions with the smaller P3′ valine (Fig. 4b). From the model, it is apparent that Val309 (P3′) has less optimal complementarity for the broad S3′ pocket compared to the bulky aromatic Phe309 (P3′) of SARS-CoV-1. The smaller hydrophobic side chain may be needed to accommodate binding to S3′ in the presence of the spatially adjacent P1′ Ser. In SARS-CoV-1, P1′ is a uniquely observed glycine (Supplementary Fig. 1b), providing the main chain torsion and lack of a side chain needed to be sterically compatible with the bulkier phenylalanine occupying the S3′ subsite^28^. Regardless, the potential buried surface of adjacent SARS-CoV-2 P1′ Ser and P3′ Val provides noncovalent interactions, presumably sufficient to facilitate the multivalent prime side subsite binding along with Phe305 (P2) to promote cleavage^29^. Further, His41 Nε2 is readily positioned inline and within hydrogen bond distance of the P1′ main chain nitrogen, supporting its general base role in leaving group protonation during acylation and in keeping with recent QM/MM studies^30^. In addition, we note the P1′ substitution of a serine as described above places its side chain hydroxyl adjacent and within hydrogen bonding distance to His41 Nε2.

The side chain amide of Asn142 also appears to be a point of conformational plasticity: in the acyl-enzyme intermediate and product complexes, with nothing bound in S2′, it is swung towards that subsite, however, in the ES complex with occupied S2′, this rotamer would be sterically prohibited and instead is redirected to stack over the P1 Gln306 in the SARS-CoV-1 ES complex structure (Fig. 4a), potentially reinforcing binding of this key subsite and ensuring maximal substrate affinity only when S2′ is occupied, and in turn disfavoring autoinhibition by C-terminal product. Finally, we also observe some small conformational differences in substrate binding between the SARS-CoV-1 ES complex and our structures here. These include the main chain atoms and rotameric state of the Val303 (P4) side chain and adjacent Met165 side chain upon which it packs, potentially perturbed by the amide side chain rotamer and main chain contacts of Gln189 in the SARS-1-CoV ES complex structure.

### Implications for drug discovery

SARS-CoV-2 M^pro^ is a major focus of antiviral drug discovery to treat COVID-19. The structures reported here provide critical information on targeting the mechanistic features and active site structure, as well as a variably exposed pocket at the dimerization interface described below.

**Fig. 5:**
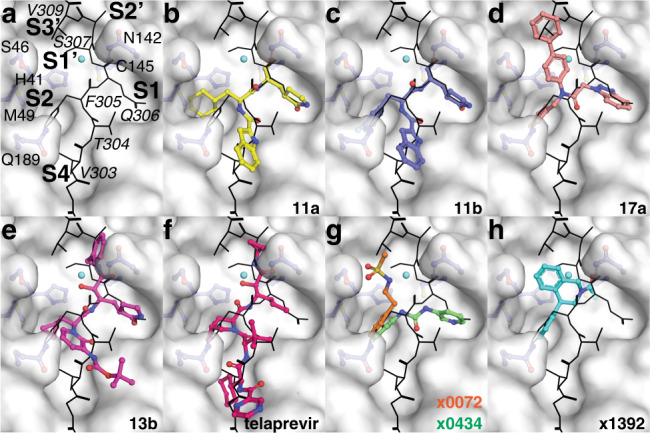
M^pro^ inhibitor binding in relation to the enzyme–substrate complex model. The surface in each panel is that of chain B of the acyl-enzyme structure. The C-terminal autocleavage site enzyme–substrate complex model for SARS-CoV-2 (see Fig. 4b) is shown in black lines. The protomer B active site binding pockets (S1, S2, S4, S2′, and S3′) and bound B′ substrate residues (italics) are labeled in panel **a. b**–**h** Superposed drugs are shown in colored cpk representation with published names provided for each. PDB accession codes: 11a—6LZE, 11b—6M0K, 13b—6Y2G, telaprevir—7C7P, x0072—5R7Y, x0434—5R83, and x1392—5RFT. Drawings for each inhibitor can be found in Supplementary Fig. 8.

The M^pro^ active site is necessarily malleable to accommodate binding of the 11 endogenous cleavage targets. In particular, the S2 subsite is significantly altered when bound to the N-terminal autoprocessing sequence with Leu (P2), as observed in complex with a SARS-CoV-1 M^pro^ H41A mutant^28^ (referred to as Leu-S2 like) compared to that captured here in complex with the wild-type SARS-CoV-2 C-terminal autoprocessing sequence (referred to as Phe-S2 like). Echoing the substrate diversity, varied hydrophobic substituents in previously characterized inhibitors have been found to bind the S2 site^7,8,10,31^. For example, designed peptidomimetic covalent aldehyde inhibitors 11a and 11b differ only in their P2 substituent with cyclohexyl or 3-fluorophenyl moieties, respectively (Fig. 5b, c and Supplementary Fig. 8). The cyclohexyl group resembles the binding of leucine, stacking with the His41 side chain, and induces a Leu-S2-like orientation of Met49. Conversely, the 3-fluorophenyl of 11b superposes near perfectly with Phe305 (P2) in our structure with a S2 subsite correspondingly in a Phe-S2 conformation. Both are promising lead inhibitors with IC_50_ values ~0.05 μM supporting drug design strategies targeting both S2 site conformations. Given the shared Phe (S2) and Phe (S3′) binding sites in the SARS-CoV-1 ES complex (Fig. 4a), and observation here that this site is preformed even in the absence of prime side residues, exploring the S3′ pocket represents a promising approach to improve inhibitors binding the Phe-S2 site. The most active noncovalent inhibitor of SARS-CoV-1 or -2 M^pro^ reported to date, compound 17a, is a derivative of a compound observed to bind both the Phe-S2 and S3′ sites of SARS-CoV-1 M^pro^ (refs. ^32,33^). In absence of an experimental structure, we docked 17a to the active site of the SARS-CoV-1 ES-like complex (PDB 5B6O). Superposing also with the SARS-CoV-2 ES complex model shows that one of the phenyl biaryl groups is likely to occupy the S3′ subsite, overlapping the position of SARS-CoV-1 Phe309 (P3′) or the equivalent SARS-CoV-2 Val (P3′; Fig. 5d). We note the only substitution between SARS-CoV-1 and -2 M^pro^ in the S3′ site—A46S—is in close proximity to the phenyl biaryl and the Ser46 hydroxyl could be a unique site to engage for further development. Thus, the structures here in complex with the C-terminal sequence provide a template for structure-based design of inhibitors targeting the Phe-S2 and S3′ sites, which could not be rationally designed using M^pro^ structures with the Leu-S2 active site conformation.

To this end, we have analyzed the SARS-CoV-2 M^pro^ active site structures of the crystallographic fragment screening effort by Walsh and colleagues^5^ that identified 71 noncovalent and covalent binding small molecules. Fragments were screened by soaking crystals with the same form as the substrate-free wild-type structure here. In these crystals, the empty active site resembles the more open Phe-S2 like conformation seen in complex with the C-terminal autocleavage site. Functional groups from both noncovalent and covalent bound fragments were observed to occupy the S2 subsite. Flexibility in S2 to accommodate binding was observed for some fragments, but the vast majority stabilized the Phe-S2 like conformation, with an aromatic functionality repeatedly observed to form hydrophobic interactions with Met49, mimicking the Phe305 (P2) interaction observed here (Fig. 5g, h). The preference for the Phe-S2 binding fragments could suggest that, although inducing the Leu-S2 like conformation was possible within the crystal, the limited binding interface of the small fragments used coupled with the initial Phe-S2 like starting structure of the substrate-free crystals used for soaking could skew the resulting binding toward this active site conformation. Several S2 site binding fragments also bind the S3’ site (Fig. 5g, h). Further to the discussion above, these fragments could represent promising starting points for development, especially by combining with those observed to bridge S2 with other subsites, for example, x0434 with overlapping S2 bound benzyl ring and a pyridine ring binding the S1 subsite (Fig. 5g).

The structures presented here also provide information on active site solvation, the consideration of which is valuable for drug design. In addition to the well-ordered active site waters we observe, which are displaced by the C-terminal substrate (Fig. 2b), we also describe a putative deacylating water observed in both the acyl-enzyme intermediate and the empty active site protomer of the C145A mutant product complex (Fig. 2c). Interestingly, structures of alpha-ketoamide inhibitors, including 13b (ref. ^7^; Fig. 5e), and hepatitis C antivirals boceprevir (PDB 6WNP) and telaprevir (PDB 7C7P; Fig. 5f), show they position carbonyl oxygens superposing with both this catalytic water and the one occupying the oxyanion hole, allowing two direct hydrogen bond interactions with the catalytic center. Notable for boceprevir and telaprevir, the P1 cyclobutyl or propyl groups do not fill the S1 site, and two waters are present overlapping with the waters we observe displaced by the C-terminal Gln (P1) side chain amide deep in the S1 pocket (Fig. 5f). Extending the P1 moieties to displace these waters to bulk solvent could be a means to improve binding. In addition, and following on our prior discussion, we also note that for these promising alpha-ketoamides, which promote the Phe-S2 conformation of SARS-CoV-2, an extension of their P1′ phenyl, amide, or cyclopropane groups into the adjacent S3′ site would also be a possible design strategy for improved potency.

**Fig. 6:**
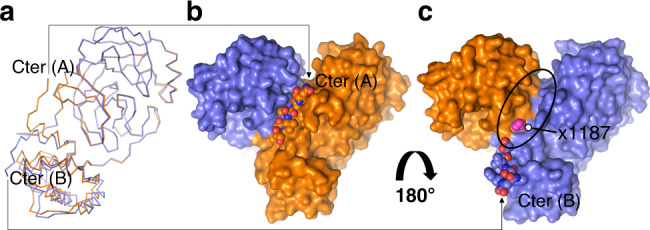
Captured alternate SARS-CoV-2 M^pro^ C-terminal conformations can inform drug discovery. **a** Superposition of SARS-CoV-2 M^pro^ acyl-enzyme intermediate protomers determined here with chain A and chain B in orange and blue, respectively. The alternate C-terminal orientations—labeled Cter (A) and (B)—observed reveal a druggable pocket at the dimerization interface. Arrows connect to corresponding C-terminal orientation in (**b**) and (**c**). **b** The C-terminus of chain A (orange VdW representations) is packed at and stabilizes the dimerization interface (blue and oranges surfaces), an interaction typical of the mature dimer. **c** In the acyl-enzyme and product complexes, chain B redirects its C-terminus ~180° (blue VdW representations) as also shown in (**a**), allowing capture within the active site cleft of a neighboring dimer in the crystal, with the extended peptide binding groove at the dimerization site now exposed (delineated by black ellipse). A recent structure-based fragment screen found several small molecules bound within this region including compound x1187 (magenta spheres; PDB 5RFA).

Finally, our structures also define a distinct binding site exposed due to the alternate positions of the C-terminal autocleavage sequence (Fig. 6a). In the mature enzyme here and in prior structures, Ser301–Gln306 typically pack at the dimerization interface, with Phe305 buried in a hydrophobic pocket defined by Phe8, Pro9, Ile52, Phe294, and the Arg298 propyl moiety of the same chain (Fig. 6b). This region is critical to dimerization and enzymatic activity, and many mutations affecting both map to this site (for review of these see^12^), including mutation of Pro9 to threonine (P9T) identified and characterized here, which shows significantly diminished dimerization and activity (Supplementary Figs. 2–4). In the catalytic snapshots captured here, when the C-terminal autocleavage site is inserted into a neighboring dimer active site, this pocket becomes more solvent exposed (Fig. 6c) and is also modulated by the movement of domain III helix J (harboring Phe294 and Arg298; Supplementary Fig. 6a). Protein–protein interaction interfaces are being increasingly targeted for drug discovery^34^, and the essential role of oligomerization in M^pro^ activity suggests that targeting of the dimerization interface with small molecules that could inhibit self-association or interfere with the inter-subunit allosteric regulation of enzymatic activity represents a promising approach. In validation of this site as druggable, Walsh and colleagues identified two small molecules from a crystallographic fragment screen that were found to bind deep into the pocket^5^ (Fig. 6c).

In this context, the M^pro^ structures presented here with the C-terminal autocleavage site bound as an acyl-enzyme intermediate or product form provides a C-terminal substrate-bound active site conformation that can be used to delineate atomic details of the mechanistic pathway, and optimize current inhibitor hits and design highly potent, novel M^pro^ inhibitors. We are currently exploring these drug design strategies with our recently described in silico deep docking methodology^35^.

## Methods

### Cloning, protein production, and purification

The gene encoding full-length SARS-CoV-2 M^pro^ with an additional N-terminal AVLQ and C-terminal GPHHHHHH was ordered from Twist Bioscience codon optimized for expression in *Escherichia coli* (Supplementary Table 3). The gene was cloned between the BamHI and XhoI restriction sites of plasmid pGEX-6P-1 (Supplementary Table 3) for expression of the protein with native N- and C-termini^11^. Mutant C145A was generated using QuickChange site-directed mutagenesis (Supplementary Table 3). Mutant P9T was a point mutant from cloning the full-length gene. Sequences were confirmed by DNA sequencing.

Protein expression was carried out in *E. coli* BL21 (DE3). Cells were grown at 37 °C in LB media supplemented with 0.1 mg/mL ampicillin. At OD_600_ ~1, the temperature was reduced to 16 °C, and protein expression was induced with addition of IPTG to 1 mM. Cells were harvested after 5 h, resuspended in lysis buffer (50 mM Tris pH 8, 300 mM NaCl, 1% triton-X100, 10 mM MgCl_2_, and 0.01 mg/mL DNase I), and lysed with an Avestin Emulsiflex C5. The lysate was centrifuged at 50,000 × *g* for 45 min and the soluble protein was loaded onto gravity flow column packed with 5 mL HisPur Ni-NTA resin (ThermoFisher Scientific) equilibrated in the lysis buffer with 20 mM imidazole. The column was washed with ten column volumes of equilibration buffer, ten column volumes of the buffer with 40 mM imidazole, and eluted with 50 mM Tris pH 8, 300 mM NaCl, and 200 mM imidazole.

For the wild-type and P9T mutant protein, which cleave off the N-terminal GST tag during expression to leave the native N-terminus, the eluate was concentrated by ultrafiltration (Amicon Ultra-30; Millipore Sigma) and the buffer was exchanged into 50 mM Tris pH 8, 300 mM NaCl, and 1 mM DTT to a final volume of 3 mL. The C-terminal His-tag was removed with HRV 3C (Millipore Sigma) incubated overnight at 4 °C. Uncleaved M^pro^ and the His-tagged HRV 3C were removed with a 0.5 mL HisPur Ni-NTA resin before further purification by gel filtration chromatography with a Sephacryl S-200 HR 16/60  column (GE Healthcare) equilibrated in 50 mM Tris pH 7.4, 1 mM EDTA, and 1 mM DTT.

Because the C145A mutant is inactive, wild-type, His-tagged M^pro^ was added to the eluate from the initial IMAC step at a 40:1 ratio, and the mixture was dialyzed overnight against 50 mM Tris pH 8.0, 300 mM NaCl. The retentate was then incubated sequentially with 0.5 mL Glutathione Sepharose resin (GE Healthcare) equilibrated in dialysis buffer and 0.5 mL HisPur Ni-NTA agarose resin equilibrated in the same buffer with 20 mM imidazole to remove the GST tag and His-tagged wild-type M^pro^, respectively, while the flow through and washes were collected. The C-terminal His-tag was removed with HRV 3C before further purification by gel filtration chromatography with a Superdex 200 Increase 10/300 GL column (GE Healthcare), as described above.

All proteins were concentrated to 10 mg/mL and frozen in liquid nitrogen for storage at −80 °C until needed.

### Analysis of protein quaternary structure

The molar masses of wild-type M^pro^ and the P9T variant were determined at 22 °C with a size-exclusion chromatography system equipped with a Superdex 200 HR 10/300 GL column (GE Healthcare), an Agilent 1100 series HPLC pump and UV detector (Agilent Technologies), a Dawn Heleos II 16-angle light-scattering detection module and an Optilab T-rEX differential refractometer (Wyatt Technology). The mobile phase was 50 mM Tris pH 7.3, 150 mm NaCl, and the flow rate was 0.4 mL/min. Data acquisition and analysis were achieved with Astra 6 software platform provided by Wyatt. The light-scattering detectors were normalized with monomeric bovine serum albumin (Sigma-Aldrich). A 100 μL aliquot of each protein (2 mg/mL) was injected into the column at a time, and the column was washed with at least one column volume between samples. The protein absolute molecular mass was calculated assuming a specific refractive index increment (*δη*/*δc*) value of 0.185 mL/g and a theoretical extinction coefficient of 0.973 mL/mg/cm.

BioSAXS data were collected with in-house X-rays (1.54 Å) and a Rigaku bioSAXS-2000 (Rigaku Corporation). Scattering profiles of purified wild-type SARS-CoV-2 M^pro^ were collected at 9.63, 4.82, 2.41, and 1.20 mg/mL and the P9T M^pro^ variant at 24.49, 12.25, 6.12, 3.06, 1.53, and 0.77 mg/mL. Twelve consecutive frames of 5 min in length were collected for each profile, corrected by subtracting the background scattering of the dialysis buffer (50 mM Tris pH 7.4, 1 mM DTT, and 1 mM EDTA), and normalized in concentration. Processing up to this point was carried out using SAXSLab (Rigaku Corporation). Further processing was performed with components of the ATSAS software package^36^. An extrapolated 0.00 mg/mL curve was generated for each of the samples. For the wild-type M^pro^ sample, the crystal structure of the M^pro^ dimer (PDB ID 6M03) was compared to the experimental data using CRYSOL^37^. For the P9T M^pro^ sample, chain A of the same structure was compared to the experimental data, with rather poor fit. As this result may stem from a slightly less restrained state of M^pro^ as a monomer, SREFLEX^38^ was used to allow for more flexible fitting of the PDB to the experimental data. To ensure that NaCl and DMSO were not affecting the dimerization state of M^pro^, bioSAXS data for various samples of 1 mg/mL M^pro^ with the addition of NaCl and DMSO were collected, and processed as above. OLIGOMER^39^ volume fraction analysis was used, with the 0.00 mg/mL extrapolated curves used as a basis for the dimer and monomer fractions. Full SAXS sample details, data collection parameters, software, structure parameters, and modeling statistics are listed in Supplementary Table 2.

### Enzymatic activity

The protease activity of recombinant wild-type M^pro^ was assayed at 27 °C with the FRET-based peptide substrate (MCA)AVLQ/SGFRLys(Dpn)-Lys-NH_2_ (GL Biochem, Shanghai) essentially as described^7,8,31,40^. The fluorescence of 7.5 μL aliquots of this substrate in 50 mM Tris buffer, 2 mM EDTA, pH 7.3, and 10% DMSO was monitored with a BioTek Synergy H4 microplate reader (330 nm excitation, 390 nm emission, and 9 nm slit band widths) for 3 min immediately before addition of 7.5 μL of enzyme in the same buffer to start the reactions. For enzymological characterization the final M^pro^ concentration was 100 nM, while that of the substrate spanned the range between 0.5 and 100 μM. The initial rates of reaction, collected in triplicate at each substrate concentration, were determined from the linear regions observed during the first 3 min of each reaction. After correcting these values for the inner filter effect and converting to units of cleaved product as a function of time (i.e., μM/s) using a calibration curve constructed with (MCA)-AVLQ, these initial reaction rates were then subjected to nonlinear, least squares regression analysis with the Michaelis–Menten equation using the program OriginPro (OriginLab Corp., Northampton MA) to determine the kinetic parameters *K*_M_ and *k*_cat_ and a Hill coefficient *n*, assuming a 100% active enzyme. Similarly, the dose-dependent inhibition of enzyme activity by Carmofur (Cayman Chemicals) was assayed to confirm that the recombinant M^pro^ behaves, as described in the literature. For this assay, the enzyme was incubated with different concentrations of Carmofur for 30 min before mixing with the substrate solution to monitor the residual activity also in at least triplicate. For this assay, the final enzyme and substrate concentrations were 30 nM and 20 μM, respectively, while that of Carmofur spanned the range from 100 nM to 30 μM.

### Crystallization and structure determination

Crystals of the wild-type acyl-enzyme or C145A mutant product complexes were obtained by sitting drop vapor diffusion using 0.8 μL of protein (~10 mg/mL) and 0.8 μL reservoir containing 0.1 M MES pH 6, 10–16% PEG 3350, and 5% MPD. Crystals were cryoprotected by increasing the PEG 3350 concentration to 35% prior to freezing in liquid nitrogen. For the wild-type acyl-enzyme crystals, the cryoprotectant solution also included 2% DMSO. Wild-type substrate-free crystals were obtained as above from a reservoir containing 0.1 M MES pH 6.5, 15–20% PEG 3350, with streak seeding used to obtain diffraction quality crystals. Diffraction data were collected at 100 K on beamline 23-ID-B at the Advanced Photon Source (wild-type acyl-enzyme and substrate free; 1.03317 Å wavelength), or on a Rigaku MicroMax 007 HF generator equipped with Osmic VariMax optics and a Dectris Pilatus3 R 200 K detector (C145A mutant; 1.5417 Å wavelength). Both wild-type acyl-enzyme and C145A mutant product complex crystals belong to space group *C*2 with isomorphous unit cell dimensions and two molecules in the asymmetric unit  (Supplementary Table 1). The wild-type substrate-free crystals also belong to space group *C*2 but with different crystal packing and only one monomer in the asymmetric unit  (Supplementary Table 1). The wild-type acyl-enzyme and substrate-free data were processed using xia2 (ref. ^41^) and XDS^42^, and the C145A mutant with the CrysAlis Pro software suite (Rigaku Inc.). Data reduction was carried out using Aimless^43^ as part of the CCP4 package^15^. The wild-type acyl-enzyme and product data exhibited anisotropy as assessed with the Diffraction Anisotropy Server^44^. Both non-truncated and truncated data were used in refinement and map calculations to assist interpretation. Phasing was carried using molecular replacement with Phaser^45^ as part of the CCP4 package, using PDB 6LU7 as a search model. Sequential rounds of model building and refinement were carried out using Coot^46^, Refmac^47^, and Buster^48^. Validation of the final models was carried out using MolProbity^49^ with excellent stereochemical model statistics, see Supplementary Table 1. The wild-type acyl-enzyme intermediate model has a Molprobity score of 1.98, clashscore of 4.16 and 96.88% Ramachandran favored, and 0% Ramachandran outliers. The C145A product complex has a Molprobity score of 1.74, clashscore of 2.67 and 97.04% Ramachandran favored, and 0.33% Ramachandran outliers. The wild-type substrate-free model has a Molprobity score of 1.37, clashscore of 3.64 and 98.68% Ramachandran favored, and 0.33% Ramachandran outliers.

Secondary structure analysis was carried out using STRIDE^50^. Solvent accessible and buried surfaces were calculated using Areaimol^15^. All structure analysis and figure preparation was carried out with PyMOL (The PyMOL Molecular Graphics System, Version 2.1 Schrödinger, LLC) and Chimera^51^.

### Docking

Before docking, protein structures were optimized using Protein Preparation Wizard module (Small-Molecule Drug Discovery Suite 2019-1, Schrödinger LLC, New York, NY, USA 2019). Docking grids were centered to the C-terminal substrates. Ligands were prepared using OpenEye’s tautomers module, in order to assign the correct ionization and tautomeric form at pH 7.4 (QUACPAC 2.0.2.2. OpenEye Scientific Software, Santa Fe, NM, USA 2019). One low-energy 3D conformation was generated for each ligand, using Openeye’s omega program in classic mode^52^. Docking was performed with Glide Single Precision module^53^.

### Reporting summary

Further information on research design is available in the Nature Research Reporting Summary linked to this article.

## Supplementary information

Supplementary Information

Reporting summary

## Data Availability

Structure factors and atomic coordinates have been deposited with the protein data bank with accession codes PDB ID 7KHP, 7JOY, and 7JP1. BioSAXS data have been deposited with SASBDB with accession code SASDJG5 and SASDJH5. Other data are available from the corresponding author upon reasonable request. Source data are provided with this paper.

## Source data

Source data
