# Supplementary material for: Crystallographic structure of wild-type SARS-CoV-2 main protease acyl-enzyme intermediate with physiological C-terminal autoprocessing site
Source: Nat Commun. 2020 Nov 18;11:5877. doi: 10.1038/s41467-020-19662-4 (PMC7674412; doi:10.1038/s41467-020-19662-4)
Supplement: Supplementary file 1 — Supplementary Information [file 41467_2020_19662_MOESM1_ESM.pdf]

SUPPLEMENTARY INFORMATION

Crystallographic structure of wild-type SARS-CoV-2 Main Protease acyl-enzyme intermediate with physiological C-terminal autoprocessing site

Jaeyong Lee[1,2,4], Liam J. Worrall[1,4], Marija Vuckovic[1], Federico I. Rosell[1], Francesco Gentile[3], Anh-Tien Ton[3], Nathanael A. Caveney[1], Fuqiang Ban[3], Artem Cherkasov[3], Mark Paetzel[2,*] and Natalie C.J. Strynadka[1,*]

[1]Department of Biochemistry and Molecular Biology and Centre for Blood Research, The University of British Columbia, Vancouver, British Columbia, Canada. [2]Department of Molecular Biology and Biochemistry, Simon Fraser University, Burnaby, British Columbia, Canada. [3]Vancouver Prostate Centre, The University of British Columbia, Vancouver, British Columbia, Canada.

[4]These authors contributed equally to this work: Jaeyong Lee, Liam J. Worrall

[*]E-mail: mpaetzel@sfu.ca (MP), ncjs@mail.ubc.ca (NCJS).

Supplementary Information includes:

- Supplementary Tables 1 to 3
- Supplementary Figures 1 to 8

Supplementary Table 1: Data collection and refinement statistics.

	Acyl-enzyme (WT) PDB 7KHP	Product (C145A) PDB 7JOY	Substrate-free (WT) PDB 7JP1
**Data collection**			
Space group	C 2	C 2	C 2
Cell dimensions			
a, *b*, *c* (Å)	124.3, 80.2, 63.2	123.7, 80.3, 63.3	113.2, 52.7, 44.6
α, β, γ (°)	90, 89.6, 90	90, 90.2, 90	90, 102.7, 90
Resolution (Å)	29.69 - 1.95 (2.02 - 1.95)*,#	26.16 - 2.0 (2.07 - 2.0)#	27.2 - 1.8 (1.86 - 1.8)
R_{sym}	0.0462 (1.597)	0.1411 (0.885)	0.102 (0.499)
R_{pim}	0.0293 (0.9888)	0.0859 (0.777)	0.066 (0.418)
CC1/2	0.999 (0.412)#	0.98 (0.619)#	0.997 (0.63)
$I / \sigma I$	12.30 (0.79)#	7.71 (0.54)#	10.5 (1.4)
Completeness (%)	99.48 (99.42)	95.89 (92.56)	96.0 (84.20)
Redundancy	3.4 (3.5)	2.8 (1.8)	3.2 (2.0)
**Refinement**			
Resolution (Å)	1.95	2.0	1.8
No. reflections	44912 (4467)	40219 (3843)	22979 (2015)
R_{work} / R_{free}	0.199 / 0.240 (0.350 / 0.359)	0.217 / 0.252 (0.320 / 0.327)	0.186 / 0.219 (0.371 / 0.401)
No. atoms			
Protein	4731	4729	2361
Ligand/ion	12	0	0
Water	141	268	140
B-factors			
Protein	54.1	34.1	34.7
Ligand/ion	66.7		
Water	50.1	36.4	49.7
R.m.s. deviations			
Bond lengths (Å)	0.016	0.011	0.016
Bond angles (°)	2.03	1.58	1.99

*Values in parentheses are for highest-resolution shell.

Acyl-enzyme (WT) and Product (C145A) diffraction exhibited anisotropy as assessed by the Diffraction Anisotropy Server. The maximum resolution limit was assessed based on $I / \sigma I$ and CC1/2 in the reciprocal lattice directions.

Supplementary Table 2: SAXS sample details, data collection parameters, software, structure parameters, and modelling statistics.

	WT M^{pro}	P9T M^{pro}
**Sample Details**	SASBDB SASDJG5	SASBDB SASDJH5
Organism	SARS-CoV-2	
Protein	M^{pro}	M^{pro} (P9T)
Monomer M from chemical composition (Da)	33,797	33,801
Concentrations (mg/mL)	1.20, 2.41, 4.82, 9.63	1.53, 3.06, 6.12, 12.25, 24.49
Solvent	50 mM Tris pH 7.4, 1 mM DTT, 1 mM EDTA	
**Data Collection Parameters**		
Instrument	Rigaku BioSAXS-2000	
Wavelength (Å)	1.54	
Beam Size (mm)	1.5	
Detector	Rigaku HyPix-3000 (pixel size: 100 µm, active area: 77.5 mm X 30.5 mm)	
q measurement range (Å^-1)	0.006 - 0.650	
Radiation damage monitoring	Manual examination of each frame	
Exposure time (s)	3,600 (12 x 300)	
Sample temperature (°C)	6	
**Software**		
SAXS data reduction	SAXSLab	
Data processing	ATSAS	
**Structural Parameters**		
q range ($Å^{-1}$)	0.0124-0.604	0.0124-0.604
Guinier analysis		
$I(0)$ (cm^{-1})	2.03 +/- 0.0038	1.22 +/- 0.0015
R_g (Å)	26.94 +/- 0.08	23.94 +/- 0.04
P(r) analysis		
$I(0)$ (cm^{-1})	2.03	1.22
R_g (Å)	26.94	23.94
Porod volume ($Å^3$)	93,046	52,323
M from Porod volume (kDa)	54,732	30,778
M from Qp (kDa)	67,917	38,192
M from MoW (kDa)	72,517	41,979
M from Vc (kDa)	59,567	35,210
M from Size/Shape (kDa)	61,444	36,614
M from Bayesian Inference (kDa)	62,350	36,900
**Atomistic Modelling**		
Symmetry	P1	P1
CRYSOL analysis - dimer (6M03)		
q range for fitting ($Å^{-1}$)	0.0124-0.604	0.0124-0.604
χ^2	2.158	801.893
Predicted R_g (Å)	26.02	25.44
Vol (Å)	90,392	90,392
Ra (Å)	1.8	1.8
Dro (e $Å^{-3}$)	0	0
CRYSOL analysis - monomer (6M03)		
q range for fitting ($Å^{-1}$)	0.0124-0.604	0.0124-0.604
χ^2	479.318	64.608
χ^2 SREFLEX		15.959
Predicted R_g (Å)	24.03	23.47
Vol (Å)	45,189	40,355
Ra (Å)	1.4	1.4
Dro (e $Å^{-3}$)	0.075	0.075

Supplementary Table 3: Optimized M^{pro} gene and primer sequences used in this study.

**Optimized gene sequence including N-terminal AVLQ and C-terminal GPHHHHHH**	GCAGTCCTGCAATCTGGCTTTCGTAAGATGGCCTTCCCATCAGGTAAAGTTGAGGGATGCATGGTGCAG<br>GTTACATGCGGCACTACGACGCTTAACGGCCTGTGGCTCGACGATGTGGTTTATTGCCCACGTCATGTG<br>ATTTGCACTTCTGAAGACATGCTGAACCCAAATTATGAAGATTTACTGATTCGCAAAAGTAATCATAAT<br>TTTCTGGTACAGGCGGGGAACGTTCAACTGCGCGTCATCGGGCACTCTATGCAGAATTGCGTCCTGAAG<br>CTGAAAGTTGATACTGCGAACCCAAAAACACCAAAATATAAGTTTGTGCGCATTCAACCGGGCCAAACT<br>TTCAGTGTTTTGGCTTGTTATAACGGCAGTCCGTCGGGTGTATATCAGTGCGCAATGCGTCCTAATTTC<br>ACGATTAAGGGGTCTTTTCTCAATGGGTCCTGTGGTTCCGTTGGTTTTAATATTGACTATGATTGCGTG<br>TCATTCTGCTATATGCACCATATGGAGTTACCGACCGGAGTGCATGCCGGCACGGATCTGGAGGGCAAT<br>TTTTATGGCCCTTTTGTAGATCGTCAGACCGCCCAAGCCGCTGGTACGGATACCACCATCACCGTGAAT<br>GTTTTAGCGTGGCTGTACGCAGCGGTGATCAACGGCGACCGTTGGTTTTTGAATCGCTTTACTACAACG<br>TTAAACGATTTCAACCTCGTTGCCATGAAGTACAATTATGAACCCCTCACTCAGGATCACGTCGACATC<br>CTGGGTCCACTGTCGGCGCAGACAGGGATTGCCGTCCTGGATATGTGTGCGTCACTGAAAGAACTGTTG<br>CAAAACGGGATGAACGGCCGTACAATCCTGGGTAGTGCGCTGCTGGAGGATGAGTTTACGCCGTTCGAC<br>GTGGTCCGGCAATGTAGTGGCGTGACCTTCCAAGGTCCACATCATCACCATCATCAT
**Primer sequences**	**Primers for restriction-free cloning into pGEX-6P-1**<br>Fwd – CTGTTCCAGGGGCCCCTGGGATCCGCAGTCCTGCAATCTGGCTTTCGTAAGATGGCCTTC<br>Rev – GACCTTCCAAGGTCCACATCATCACCATCATCATTAAGAATTCCCGGGTCGACTCGAGC<br><br>**Primers for C145A mutation**<br>Fwd – CGATTAAGGGGTCTTTTCTCAATGGGTCCGCTGGTTCCGTTGGTTTTAATATTGACTATG<br>Rev – CATAGTCAATATTAAAACCAACGGAACCAGCGGACCCATTGAGAAAAGACCCCTTAATCG

a

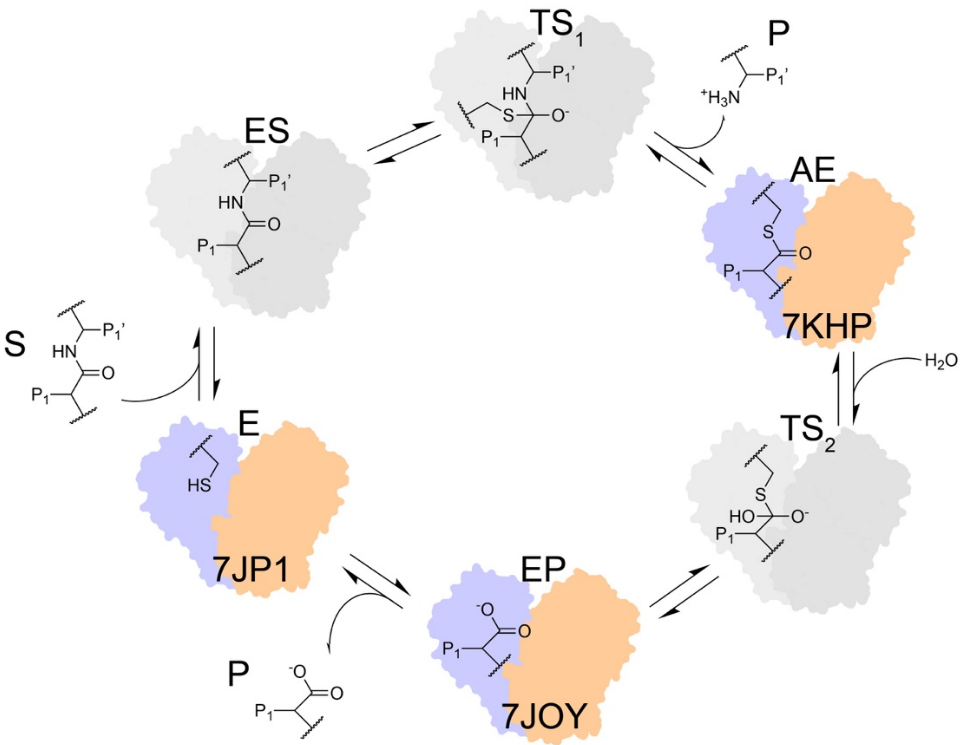

b

```
SARS-CoV-2
  P4P2     P3'
  |  |      |
TSAVLQ | SGFRKM (N-terminal auto-cleavage, nsp4 | nsp5)
SGVTFQ | SAVKRT (C-terminal auto-cleavage, nsp5 | nsp6)
KVATVQ | SKMSDV (nsp6 | nsp7)
NRATLQ | AIASEF (nsp7 | nsp8)
SAVKLQ | NNELSP (nsp8 | nsp9)
ATVRLQ | AGNATE (nsp9 | nsp10)
REPMLQ | SADAQS (nsp10| nsp11, nsp10, nsp12)
PHTVLQ | AVGACV (nsp12| nsp13)
NVATLQ | AENVTG (nsp13| nsp14)
TFTRLQ | SLENVA (nsp14| nsp15)
FYPKLQ | SSQAWQ (nsp15| nsp16)

SARS-CoV-1
  P4P2     P3'
  |  |      |
TSAVLQ | SGFRKM (N-terminal auto-cleavage, nsp4 | nsp5)
SGVTFQ | GKFKKI (C-terminal auto-cleavage, nsp5 | nsp6)
KVATVQ | SKMSDV (nsp6 | nsp7)
NRATLQ | AIASEF (nsp7 | nsp8)
SAVKLQ | NNELSP (nsp8 | nsp9)
ATVRLQ | AGNATE (nsp9 | nsp10)
REPLMQ | SADAST (nsp10| nsp11, nsp10, nsp12)
PHTVLQ | AVGACV (nsp12| nsp13)
NVATLQ | AENVTG (nsp13| nsp14)
TFTRLQ | SLENVA (nsp14| nsp15)
FYPKLQ | ASQAWQ (nsp15| nsp16)
```

Supplementary Fig. 1: **M^{pro} reaction schematic and cleavage site specificity**. **a** Reaction schematic for M^{pro} catalyzed proteolytic cleavage. S – substrate, E – enzyme, TS – transition state, AE – acyl-enzyme, P – product. Structures captured here shown in color with associated PDB IDs. **b** M^{pro} cleavage site specificity sequences (P6-P6′). SARS-CoV-1 UniProt accession number P0C6X7, SARS-CoV-2 P0DTD1. A vertical line depicts the cleavage site.

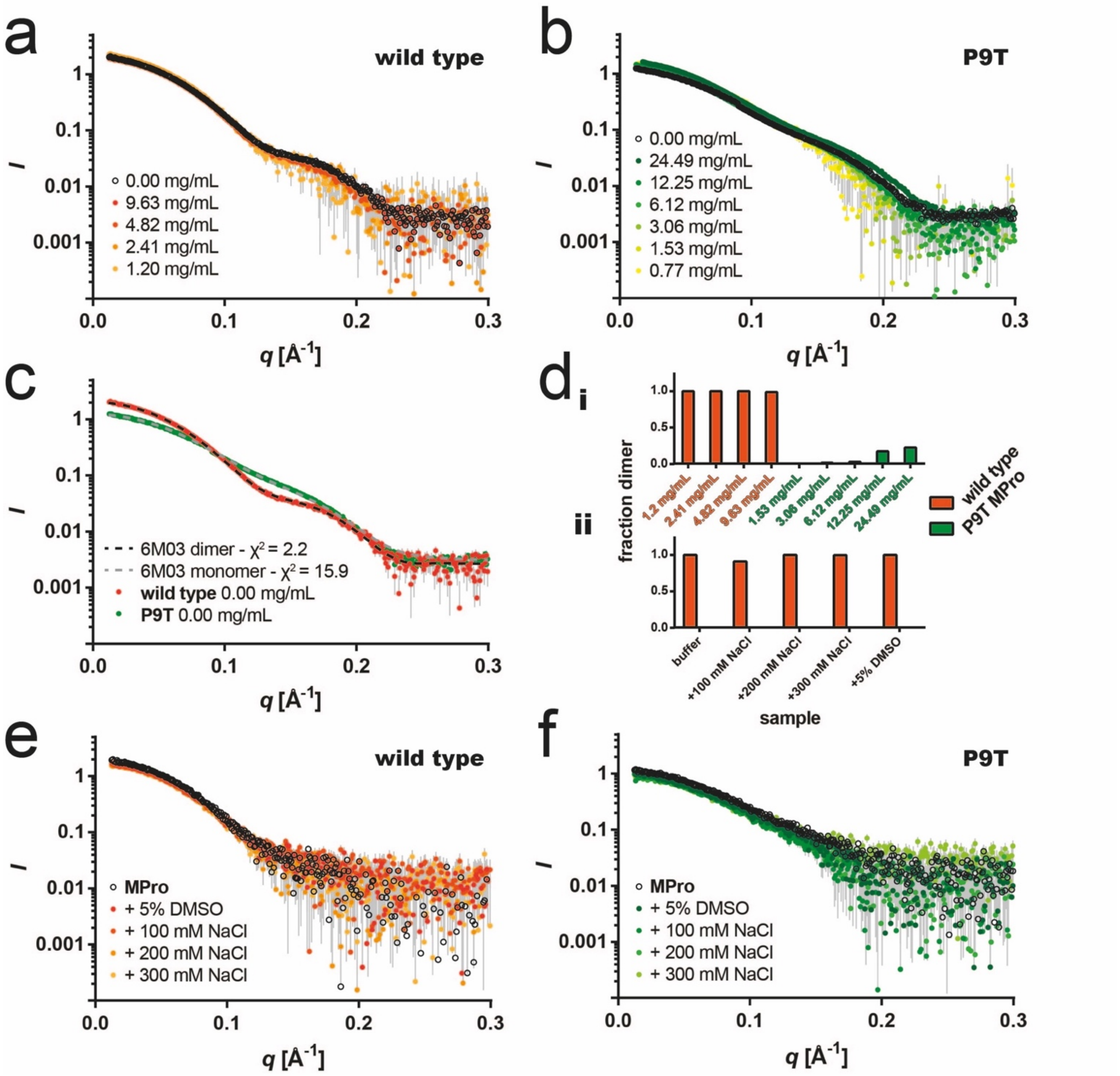

Supplementary Fig. 2: Biological Small-Angle X-ray Scattering (BioSAXS) analysis of wild-type and P9T mutant M^pro^. **a** BioSAXS data for wildtype M^pro^ at various concentrations and an extrapolated 0.00 mg/mL curve. **b** BioSAXS data for P9T M^pro^ at various concentrations and an extrapolated 0.00 mg/mL curve. **c** CRYSOL fitting of PDB dimeric and monomeric M^pro^ structures (PDB 6M03) to the 0.00 mg/mL extrapolated data. SREFLEX was used to remove potential dimer bias from the M^pro^ and allow for more flexible fitting in the case of the monomer-P9T fitting. **d** Oligomer volume fraction analysis of wild type and P9T M^pro^ using the extrapolated 0.00 mg/mL curves as basis for dimeric (wild-type) and monomeric (P9T) M^pro^. Fraction analysis of M^pro^ from **a** and **b.** (**i**) and analysis of 1 mg/mL M^pro^ with the addition of 5% DMSO and increasing NaCl up to 300 mM (**ii**). **e**,**f** BioSAXS data used in **d ii**.

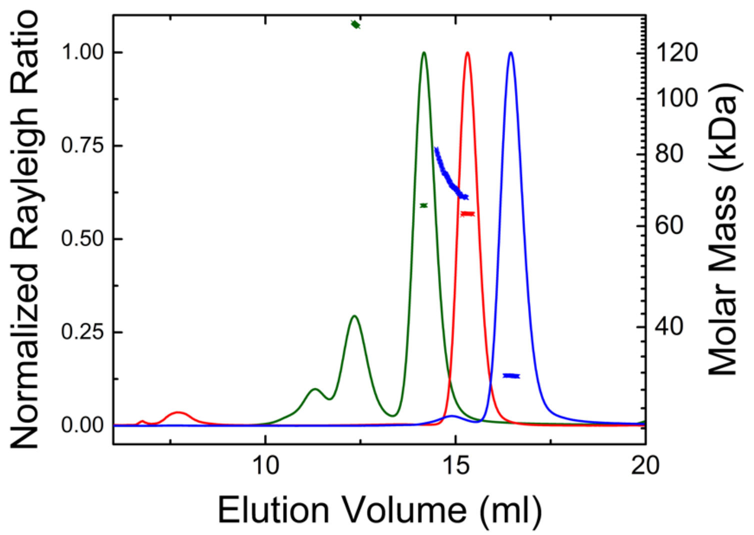

Protein	Peak	Vol_{elut} (ml)	Molar Mass (kDa)	Mass Fraction (%)
**BSA**	1	14.1	65.2	81.6
	2	12.4	134	15.4
	3	11.3	253.9	3.0
**wt-M^{pro}**	1	15.3	63.1	100.0
**M^{pro}-P9T**	1	16.4	32.9	97.7
	2	14.9	71.6	2.3

Supplementary Fig. 3: Size-Exclusion Chromatography Multi-Angle Light Scattering (SEC-MALS) analysis of wild-type and P9T mutant M^{pro}. Size exclusion chromatography elution profiles with overlaid calculated molar mass for elution peaks shown in respective colors as in table. Wild-type M^{pro} (red) elutes as a single peak with a calculated molecular mass consistent with a dimer. The P9T mutant elutes with a predominant monomeric peak with some evidence of a minor dimer species (blue). BSA (green) run for calibration and shown for comparison.

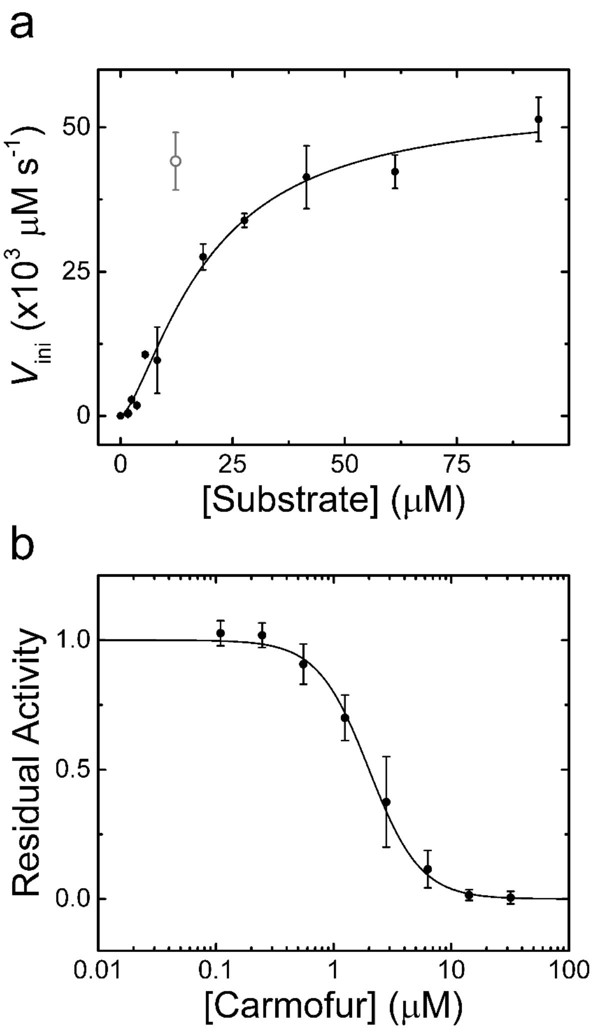

c

	WT-M^{pro}	P9T-M^{pro}
k_{cat} (s^{-1})	0.54 ± 0.04	0.019 ± 0.003
K_M (μM)	19 ± 3	36 ± 8
n	1.5 ± 0.2	1.87 ± 0.4
k_{cat}/K_M (M^{-1} s^{-1})	28000 ± 5000	500 ± 100

Supplementary Fig. 4: Proteolytic activity of wild-type and P9T mutant of SARS-CoV-2 M^{pro}. a Activity of wild-type SARS-CoV-2 M^{pro} (100 nM enzyme) shown as a plot of initial velocity verses substrate concentration, using the substrate: (MCA)-AVLQ/SGFR-Lys(Dnp)-Lys-NH_2. The datum represented in grey as a hollow circle was omitted for the non-linear regression analysis of the data to derive enzymology parameters but is shown here for completeness. Data are presented as mean values +/- SD calculated from at least three data points. **b** Dose dependent inhibition of SARS-CoV-2 M^{pro} activity by the antineoplastic agent carmofur (IC_{50} = 1.8 ± 0.3 μM, n = 2.0 ± 0.1). Assay conducted at 27 °C in 50 mM Tris buffer, 2 mM EDTA pH 7.3, 10 % DMSO with 20 μM substrate and 100 nM M^{pro}. Data are presented as mean values +/- SD calculated from at least three data points. **c** Enzymatic parameters for the wild-type SARS-CoV-2 M^{pro} and the P9T M^{pro} mutant. n = the Hill coefficient.

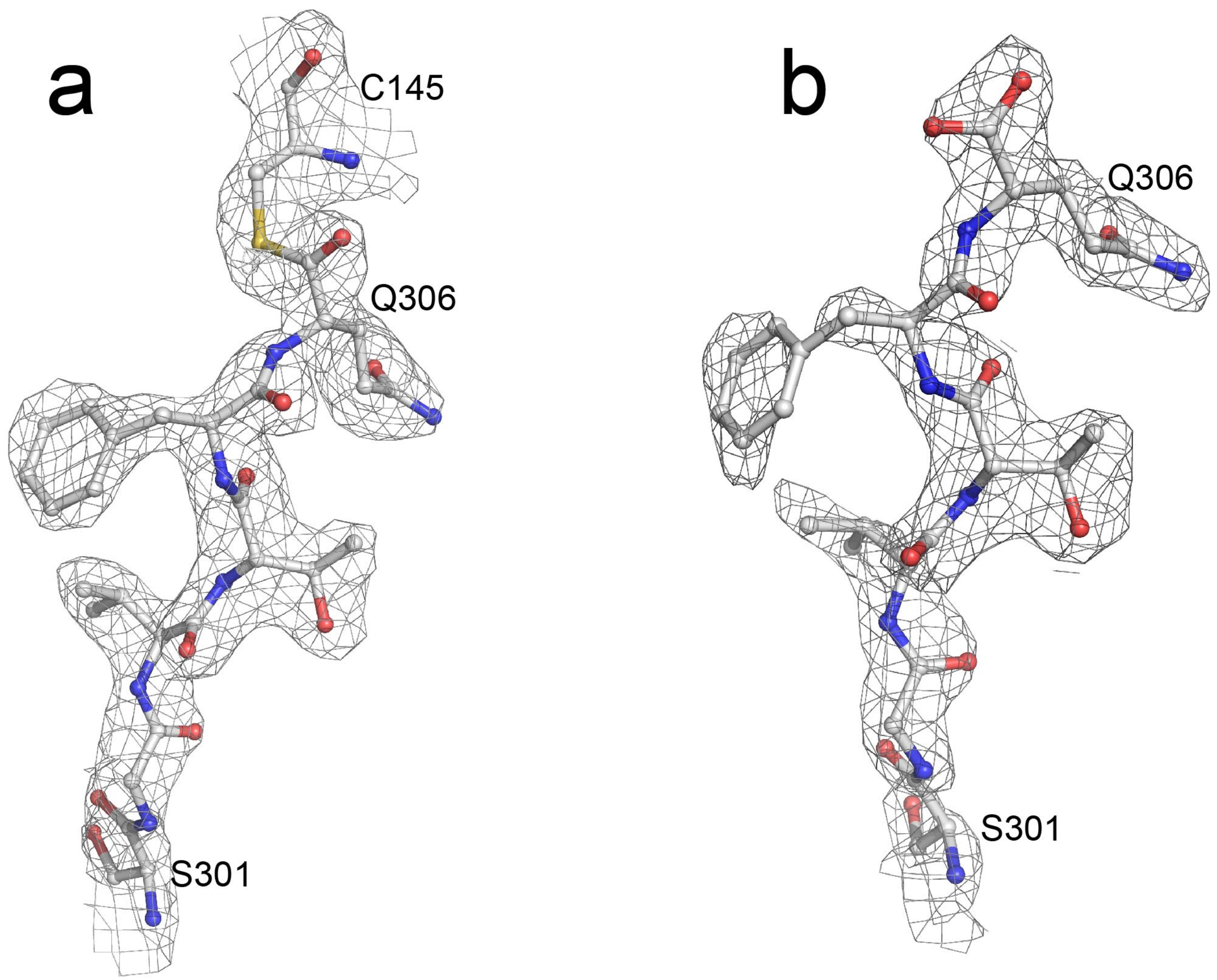

Supplementary Fig. 5: Simulated annealing 2mFo-DFc OMIT electron density maps for the active site of M^{pro} acyl-enzyme complex and product complex. a Simulated annealing 2mFo - DFc OMIT maps calculated in absence of C-terminal Ser301-Gln306 autocleavage sequence bound in active site of chain B for the M^{pro} acyl-enzyme complex. **b** As **a** for the M^{pro} C145A product complex. Map contoured at 1 σ with a 2 Å carve around the omitted atoms.

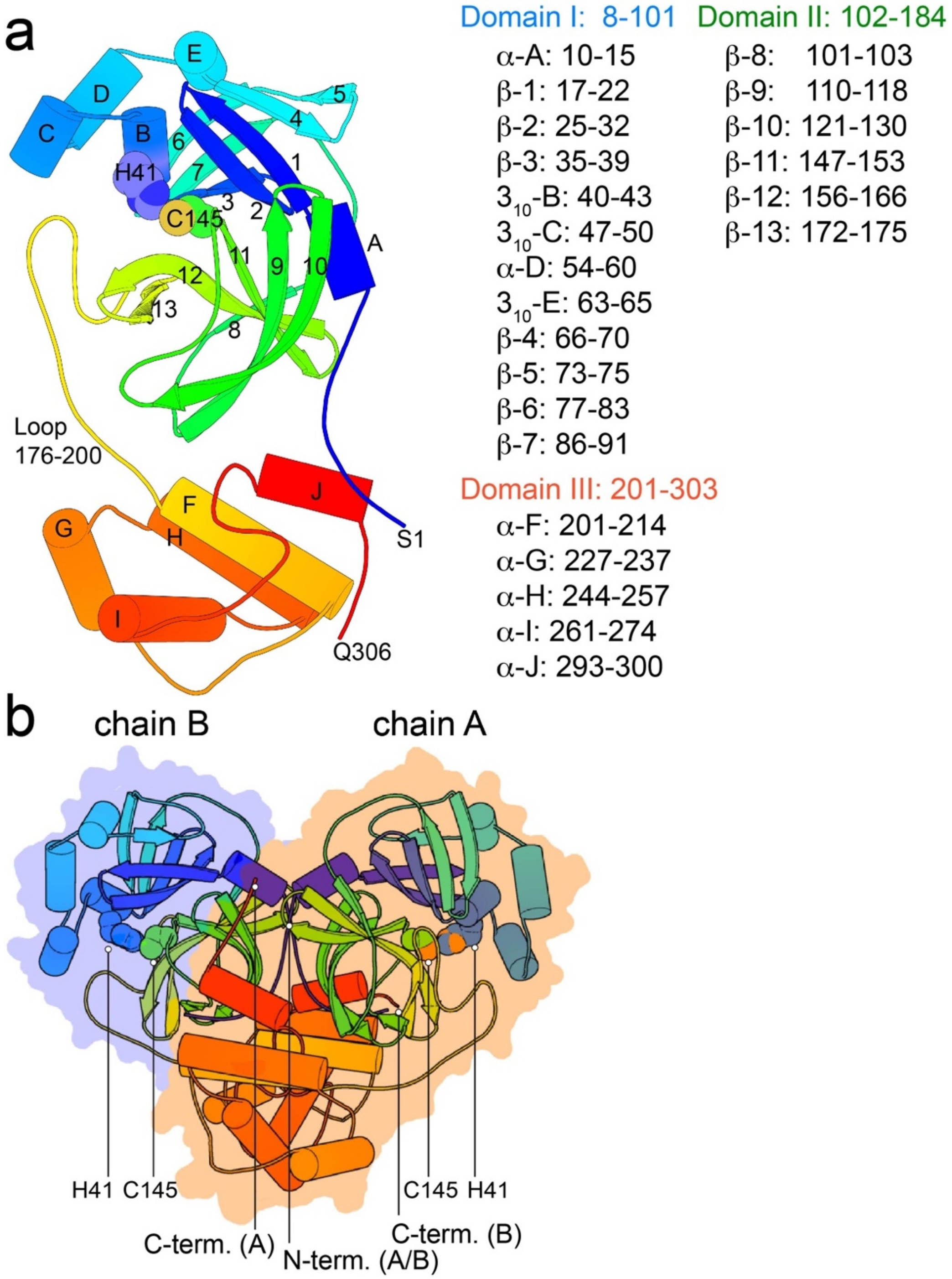

Supplementary Fig. 6: The secondary structure and dimeric structure of SARS-CoV-2 M^{pro}. a A cartoon drawing showing the secondary structural elements with helices as cylinders and β-strands as arrows. The protomer is colored spectrally from N-terminus (blue) to C-terminus (red). The amino acid residue range for domains (I-III) and the secondary structural elements are listed. **b** M^{pro} dimer. Each protomer is shown in cartoon and colored spectrally (N-terminus blue to C-terminus red). A transparent molecular surface is shown around each protomer (chain A – orange, chain B- blue).

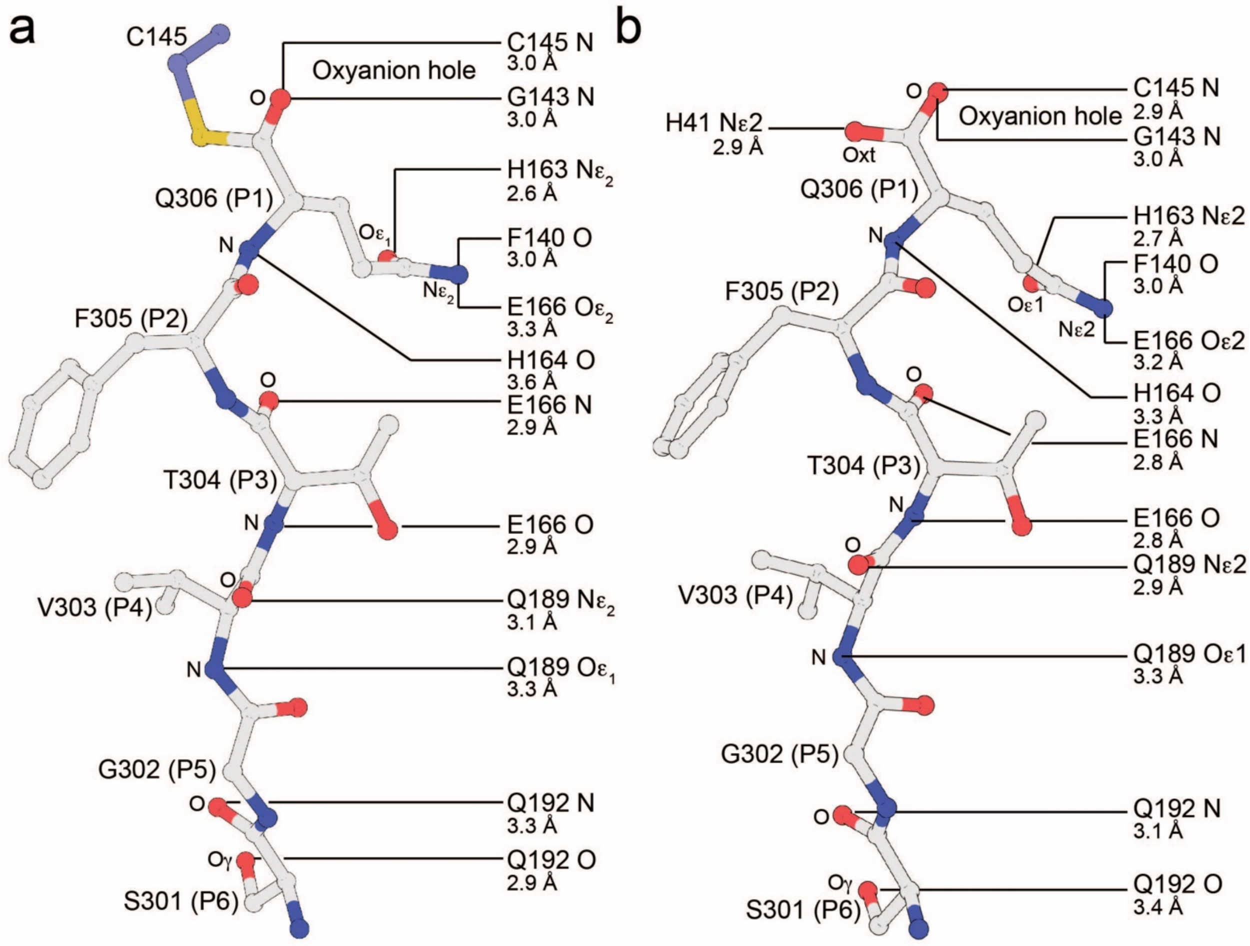

Supplementary Fig. 7: The hydrogen bonding distances observed between the C-terminus substrate of chain B′ and the catalytic groove of chain B for the acyl-enzyme (a) and product (b) complexes.

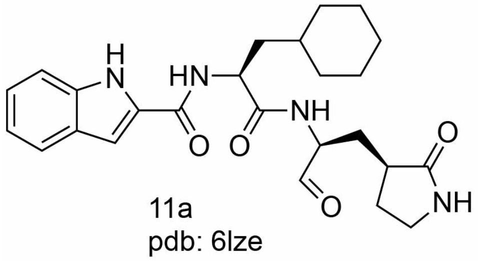

11a
pdb: 6lze

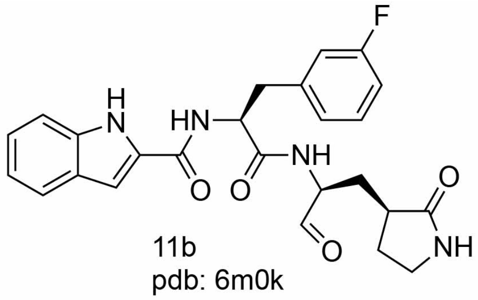

11b
pdb: 6m0k

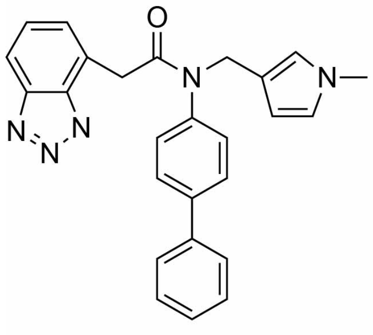

17a

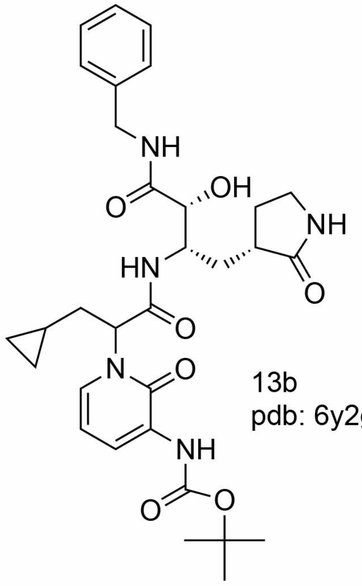

13b
pdb: 6y2g

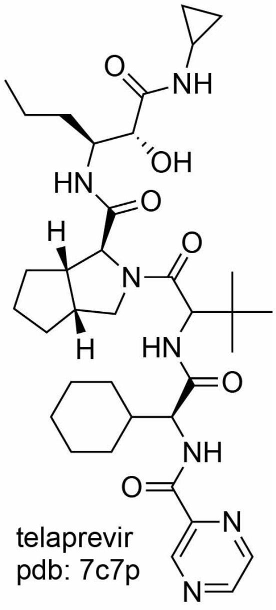

telaprevir
pdb: 7c7p

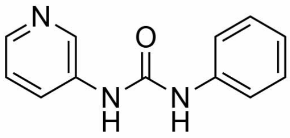

x0434
pdb: 5R83

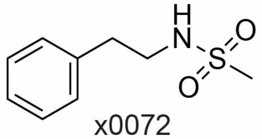

x0072
pdb: 5R7Y

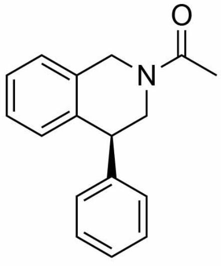

x1392
pdb: 5RFT

Supplementary Fig. 8: Chemical structures of the M^pro inhibitors depicted in Fig. 5.
